# Targets identified from exercised heart: killing multiple birds with one stone

**DOI:** 10.1038/s41536-021-00128-0

**Published:** 2021-04-09

**Authors:** Hongyun Wang, Yuling Xie, Longfei Guan, Kenneth Elkin, Junjie Xiao

**Affiliations:** 1 Institute of Geriatrics (Shanghai University), Affiliated Nantong Hospital of Shanghai University (The Sixth People’s Hospital of Nantong), School of Medicine, Shanghai University, Nantong, China; 2 Cardiac Regeneration and Ageing Lab, Institute of Cardiovascular Sciences, Shanghai Engineering Research Center of Organ Repair, School of Life Science, Shanghai University, Shanghai, China; 3 China-America Institute of Neuroscience, Beijing Luhe Hospital, Capital Medical University, Beijing, China; 4 Wayne State University School of Medicine, Detroit, MI USA

**Keywords:** Preventive medicine, Cardiac hypertrophy, Cardiovascular diseases

## Abstract

Cardiovascular diseases (CVDs) are a major cause of mortality worldwide, which are mainly driven by factors such as aging, sedentary lifestyle, and excess alcohol use. Exercise targets several molecules and protects hearts against many of these physiological and pathological stimuli. Accordingly, it is widely recognized as an effective therapeutic strategy for CVD. To investigate the molecular mechanism of exercise in cardiac protection, we identify and describe several crucial targets identified from exercised hearts. These targets include insulin-like growth factor 1 (IGF1)-phosphatidylinositol 3 phosphate kinase (PI3K)/protein kinase B (AKT), transcription factor CCAAT/enhancer-binding protein β (C/EBPβ), cardiac microRNAs (miRNAs, miR-222 and miR-17-3p etc.), exosomal-miRNAs (miR-342, miR-29, etc.), Sirtuin 1 (SIRT1), and nuclear factor erythroid 2‑related factor/metallothioneins (Nrf2/Mts). Targets identified from exercised hearts can alleviate injury via multiple avenues, including: (1) promoting cardiomyocyte proliferation; (2) facilitating cardiomyocyte growth and physiologic hypertrophy; (3) elevating the anti-apoptotic capacity of cardiomyocytes; (4) improving vascular endothelial function; (5) inhibiting pathological remodeling and fibrosis; (6) promoting extracellular vesicles (EVs) production and exosomal-molecules transfer. Exercise is one treatment (‘stone’), which is cardioprotective via multiple avenues (‘birds’), and is considered ‘killing multiple birds with one stone’ in this review. Further, we discuss the potential application of EV cargos in CVD treatment. We provide an outline of targets identified from the exercised heart and their mechanisms, as well as novel ideas for CVD treatment, which may provide novel direction for preclinical trials in cardiac rehabilitation.

Cardiovascular diseases (CVDs) have become a leading cause of death worldwide. In 2016, over three-quarters of death caused by CVD were heart attack and stroke, specifically. Several factors contribute to the increasing incidence of heart attack and stroke, including a sedentary lifestyle, cigarette smoking, and air pollution, etc.^1–6^. Thus, it is a vast and difficult challenge to prevent and treat CVDs. Exercise, however, benefits heart and reduces the risk of CVDs such as ischemic heart disease^7^, coronary heart disease^8–10^ and heart failure^11–14^. Therefore, aerobic exercise is a primary recommendation for prevention of CVD and for clinical rehabilitation. Notably, not only rehabilitation exercise training, but also voluntary physical activity significantly improves cardiac function^15,16^. Physical capacity and exercise tolerance have even been demonstrated to be a prognostic factor for the patient outcomes in CVD^14,17,18^. Strenuous exercise or high-intensity endurance exercise may induce underlying deleterious influence on cardiac function^19,20^, which is not considered here. Considerations including low physical capacity and exercise intolerance are not yet in our discussion either. In this review, exercise refers to an appropriate intensity of aerobic exercise, including treadmill running, swimming and other forms of voluntary exercise.

We thoroughly review molecular targets identified from exercised hearts as well as the mechanisms of exercise-induced cardiac protection (Fig. 1). Specific targets identified from exercised hearts include insulin-like growth factor 1 (IGF1)-phosphatidylinositol 3 phosphate kinase (PI3K)/protein kinase B (AKT) signaling, C/EBPβ-CITED4 (transcription factor CCAAT/enhancer-binding protein β-CREB-binding protein/p300-interacting transactivator with E/D-rich tail 4), cardiac miRNAs (miR-222, miR-17-3p, etc.), exosomal-microRNAs (miR-342, miR-29, etc.), SIRT1 (Sirtuin 1), and Nrf2/Mts (nuclear factor erythroid 2‑related factor/metallothioneins). Briefly, exercise promotes cardiomyocyte proliferation, physiological hypertrophy, and anti-apoptotic capacity. We also review related cell types (cardiac fibroblasts and endotheliocytes) in response to exercise. Given the potential value of extracellular vesicles (EVs) on CVDs treatment, we also discuss the benefits of exosomal-molecules in exercised hearts.

**Fig. 1:**
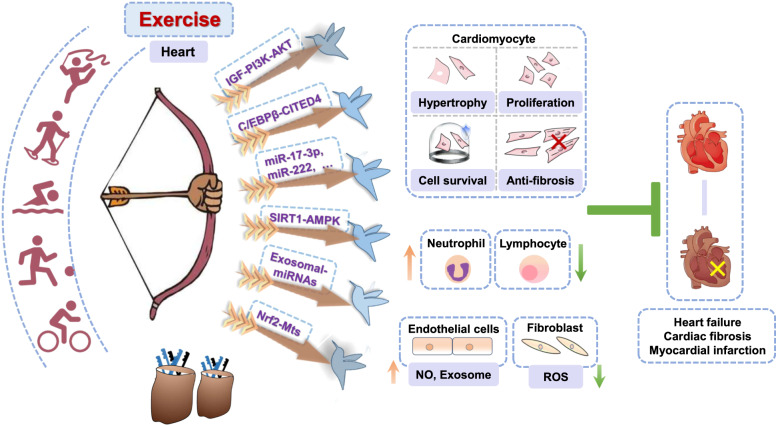
Targets identified from exercised hearts protect against cardiac disorder via multiple avenues: killing multiple birds with one stone. Specific target identified from exercised heart (IGF1-PI3K/AKT signaling, C/EBP-β-CITED4, cardiac miRNAs (miR-222, miR-17-3p, etc.), exosomal-miRNAs (miR-342, miR-29, etc.), SIRT1 and Nrf2-Mts) was ‘stone’, multiple effects of exercise on hearts were ‘birds’. Therefore, exercise protects hearts via ‘killing multiple birds with one stone’.

## Exercise reduces the risk for CVDs

An effective intervention for CVD, exercise has been extensively studied. It protects the heart against cardiac disorders, such as heart failure, myocardial infarction (MI), hypertension, and insulin resistance^17^. (1) Exercise alleviates heart failure. An action trial with more than 3000 participants found that aerobic exercise causes a significant decrease in heart failure hospitalizations as well as decreased cardiovascular events^21^. (2) Exercise protects heart against MI, inhibiting the release of cardiac proinflammatory factors, which prevents pathologic changes^22^. Mechanistically, exercise augments the anti-apoptotic capacity and is a primary mechanism for its attenuating MI development. For example, cardiac C/EBPβ reduction as well as an increase in miR-222 contributes to blocking MI-induced cell apoptosis and, thus, pathological remodeling^23^. (3) Exercise intervention reduces blood pressure (BP) and attenuates hypertension^24^. The mechanism of exercise protecting BP is mainly associated with augmented endothelial functions^25^. As we discuss in part 3, exercise actives endothelial nitric oxide synthase (eNOS) which promotes nitric oxide (NO) production, and improves endothelial functions, mediating exercise-induced BP reduction. Additionally, oxidative stress in endothelial cells is also associated with exercise-induced endothelial protection^26^. (4) Exercise improves insulin resistance, reducing the risk for CVDs, as insulin resistance is a well-studied risk factor for CVDs according to a plethora of meta-analysis and systematic reviews. Clinically, insulin resistance is considered a better predictor of CVD events than fasting glucose levels^27^. Evidently, improving insulin sensitivity is beneficial to the cardiovascular health. Aerobic exercise can significantly improve insulin resistance and mitochondrial function, itself serving as an effective strategy for the treatment of metabolic syndrome^28,29^. Of note, the age, gender as well as exercise intensity should be considered when improving insulin resistance with exercise training. Different exercise intensity in different populations can produce different effects.

First, the effect of exercise on insulin resistance is associated with the intensity of exercise. In one study, low intensity physical exercise before each night shift did not affect glucose tolerance in rotating night shift workers^30^. High-intensity interval training, however, is found to significantly improve glucose homeostasis, functional capacity and body composition in healthy individuals^31^. A recent meta-analysis demonstrates that there is no difference in the glycemic and lipid level between resistance exercise and aerobic exercise^32^. Further, aerobic exercise alone or combined with resistance exercise provides similar effects on insulin sensitivity improvement in obese adolescents^33^. Aerobic exercise and resistance exercise combined with metformin are both effective in controlling glucose level. However, Walid found that resistance exercise is better than aerobic exercise when combined with metformin to treat the type 2 diabetes^34^.

Second, the protective effects of exercise vary with age and gender. For example, the young have been found to benefit more with respect to serum lipid and glucose homeostasis than the older after high-intensity exercise^31^. Resistance exercise-induced growth hormone and IGF1 molecular weight isoform are sex dependent^35^. Interestingly, Kaitlyn et al., report that the association between the exercise intensity and insulin sensitivity is also sex dependent, as it was observed that improvement of insulin sensitivity is associated with exercise intensity in obese men, but not obese women^36^.

## Cellular adaptions in exercised hearts

**Fig. 2:**
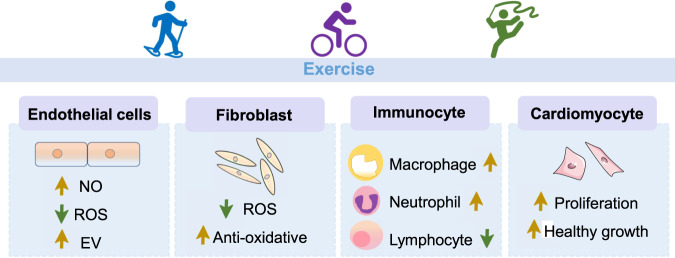
Cellular adaptions in response to exercise. In endothelial cells, exercise inhibits ROS (reactive oxygen species) production and promotes NO (nitric oxide) production as well as EV (extracellular vesicles) secretion. Exercise also inhibits ROS and improves antioxidative capacity of cardiac fibroblasts. Besides, exercise induces a rapid increase in macrophage and neutrophil number, whereas decrease in lymphocyte number. All these adaptions directly or nondirectly contributes to exercise-induced cardiomyocyte proliferation and physiological hypertrophy.

In exercise-induced cardiac protection, many types of cells are physiologically altered. We examined four of these cells (cardiomyocytes, endothelial cells, cardiac fibroblasts, and immunocytes) and evaluate their changes following exercise (Fig. 2).

### Cardiomyocyte

More energy consumption is required during exercise, leading to an increase in cardiac preload or afterload. Under this condition, cardiomyocytes tend to become physiologically hypertrophic (growth and proliferation) to match the increased demand. Adult cardiac physiological hypertrophy is characterized by an adaptive increase in cardiac mass as well as cardiomyocytes growth in length and width^37^ without collagen variation and maladaptive remodeling^38^. Many signaling pathways contribute to exercise-induced cardiac physiological hypertrophy, including IGF1-PI3K-AKT-C/EBPβ and miRNAs, which are discussed in section 3.

### Vascular endothelial cells

Moderate aerobic exercise improves vascular endothelial function via promotion of NO/vascular endothelial growth factor (VEGF) and of antioxidant enzyme. Additionally, the induction of superoxide dismutase (SOD) and peroxidase by exercise can inhibit reactive oxidative species (ROS) production in endotheliocytes^39^, and can also trigger endothelial cells to secret EVs and that influence cardiac function^40^. In the aging population, exercise is found to promote endothelial cell health by elevating CXC chemokine receptor (CXCR4)/janus kinase (JAK-2) signaling^41^.

### Cardiac fibroblasts

Fibroblasts play critical roles in extracellular matrix remodeling and cardiac repair under certain stimuli, whose activation can interact with cardiomyocytes and maintain the cardiac microenvironment^42^. When activated, the fibroblast converts to a myoblast, contributing to cardiac repair or fibrosis^43^. Recent evidence demonstrates that exercise activated the nuclear factor erythroid 2‑related factor (Nrf2) signaling in fibroblasts, subsequently elevating the expression of metallothioneins (Mts, including Mt1 and Mt2) and causing cardiomyocyte physiological hypertrophy^44^.

### Immunocyte

Immunocytes are involved in exercise-induced cardiac protection. Clinical investigation indicates that circulating neutrophils and monocytes increase after exercise by promoting lymphocyte translocation to potential antigen sites in the heart. By contrast, exercise causes a significant reduction in lymphocytes number^45^.

## Pivotal targets identified from exercised heart

### IGF1-PI3K-AKT signaling pathway

The IGF1-PI3K-AKT pathway is extensively studied with respect to exercise-induced cardiomyocytes growth and hypertrophy^46^. IGF1 can be activated by exercise, and then binds to its receptors (IGF-1R) on cardiomyocytes, causing intracellular PI3K/AKT pathway activation. During IGF-1R recycling, tumor susceptibility gene 101 (Tsg101) is found to interact with family-interacting protein 3 (FIP3), whose combination further increased recycling. Thus, Tsg101 is identified as a novel target in exercised heart, further increasing cardiomyocyte hypertrophy. Treadmill exercise training significantly promoted the expression of Tsg101 in mice and Tsg101-deficient mice counteracted exercise-induced cardiac physiological hypertrophy^47^. Additionally, Akt is a target of PI3K and has 3 isoforms (AKT1, AKT2, AKT3). Of these, AKT1 is required for exercise-induced cardiac physiological hypertrophy and growth^48^. In cardiac-specific AKT1 transgenic mice, an 80% increase in AKT activity caused 2.2-fold increase in heart weight compared with that of control group^49^.

### C/EBPβ

**Fig. 3:**
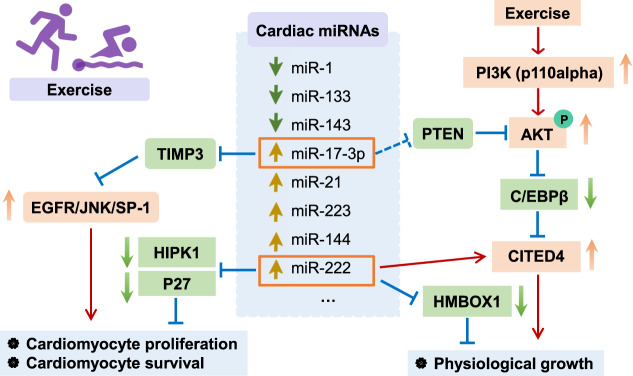
MiRNAs in exercise-induced cardioprotection. The mechanism of PI3K/AKT, C/EBP-β-CITED4, and cardiac miRNAs (miR-222, miR-17-3p) in exercise-induced cardiomyocyte hypertrophy, proliferation, and survival.

C/EBPβ, the transcription factor CCAAT/enhancer-binding protein β, is downstream of PI3K/Akt signaling, contributing to exercise-induced cardioprotection. Endurance exercise training can induce cardiomyocyte growth and proliferation, via AKT activation, and also suppresses C/EBPβ production. Inhibition of C/EBPβ in cardiomyocytes caused an increase in cell size and number, indicating that the C/EBPβ deficiency promoted the cardiomyocytes growth and proliferation^50^. Two axes are involved in C/EBPβ pathway in response to exercise: the C/EBPβ-CITED4 axis and the C/EBPβ-GATA4 axis (Fig. 3).

#### C/EBPβ-CITED4 axis

Exercise-induced CITED4 upregulation can induce cardiomyocyte growth at basal condition^50^. CITED4 induces physiological hypertrophy (~25%-fold of increase in heart mass) without causing pathologic changes in male and female mice^51^, whose upregulation significantly promoted an increase in cell surface area, proliferation markers expression (Ki67 and EdU), and cell number^52^. Mechanistically, cardiac CITED4-induced hypertrophy is also associated with upregulation of neuregulin-1 (NRG1)^52^.

#### C/EBPβ-GATA4 axis

GATA4 is a key regulator of cardiac hypertrophy and cardiomyocyte viability^53^. Exercise training causes a significant increase in cardiac GATA4 expression in MI in mice, whose elevation is cardioprotective^54^. Mechanistically, GATA4 deficiency upregulates the expression of proapoptotic proteins (caspase12 and Bcl6), alters FGF1 (fibroblast growth factor 1) and EGFR expression, leading to a reduced hypertrophic response^53^.

### Cardiac microRNAs

MicroRNAs, small noncoding RNA molecules (~22 nucleotides), regulate gene expression in exercise-induced cardiomyocyte proliferation and growth. With RNA arrays or qRT-PCRs, several miRNAs have been identified from the exercised heart, such as miR-222, miR-17-3p, miR-21, and miR-124^55,56^. Here, we review the roles and mechanisms of miR-222 and miR-17-3p in exercise-induced cardiac physiological hypertrophy (Fig. 3).

#### MiR-222

MiR-222 was initially identified as a proproliferation factor in exercised mice models. To examine the microRNA profiling, microRNA arrays have been performed and revealed an increase in miR-222 content in exercised mice, which is consistent with that of the plasma of exercised heart failure patients. The elevation of miR-222 promotes cardiomyocyte growth and proliferation (Fig. 3). Interestingly, different targeted genes of miR-222 are found to exert different roles in cardiomyocyte. For example, miR-222 induces cardiomyocyte proliferation and growth via inhibition of Kip1 (p27) and homeodomain interacting protein kinase 1 (HIPK1). Genetic deletion of HIPK1 or p27 promotes cardiomyocyte physiological hypertrophy and proliferation, both mediators of exercise-induced cardiac protection. However, Homeobox Containing 1 (HMBOX1), another downstream target of miR-222, promotes cardiomyocyte growth and hypertrophy, but has little effect on the cardiomyocyte proliferation^23^.

#### MiR-17-3p

MiR-17-92 cluster is well studied and contributes to cardiomyocyte growth and proliferation^57–59^. The miR-17-92 cluster was initially reported in cancers and disorders, whose upregulation promotes cell proliferation^60^. Following this, investigators extensively examined the role of miR-17–92 cluster in hearts using cardiac-specifically transgene or knockout mice, finding miR-17–92-promoted cardiomyocyte proliferation in postnatal and adult hearts^59^. Notably, in passenger RNA of miR-17-92 cluster, miR-17-3p has been identified as a mediator for exercise-induced cardiac growth and proliferation in mice models. In exercised hearts, miR-17-3p promotes cardiomyocyte proliferation, growth, and survival, via targeting of metallopeptidase inhibitor 3 (TIMP3), which activates epidermal growth factor (EGF) receptor (EGFR), and c-Jun NH(2)-terminal kinase (JNK)/SP-1^57^. Lastly, miR-17-3p indirectly regulates the phosphate and tension homology on chromosome ten (PTEN)/ protein kinase B (AKT) pathway, promoting cardiomyocyte cell survival.

### Exosomal-microRNAs

**Fig. 4:**
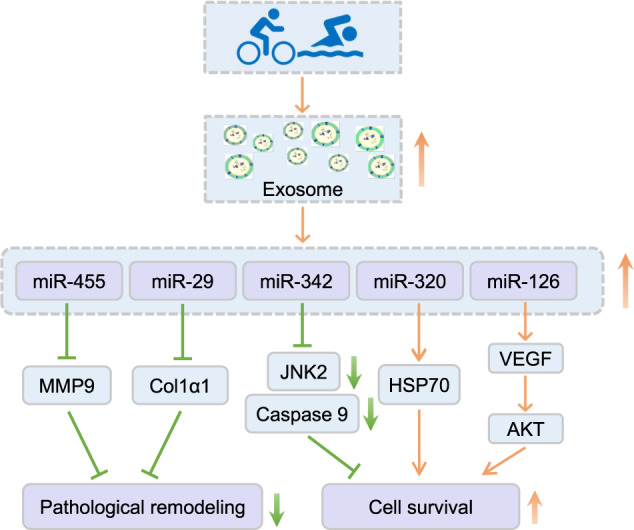
Exosomal-miRNAs identified from exercised hearts. Exercise promotes EVs and exosomal-miRNAs secretion. This figure shows the mechanism of exosomal-miR-455, miR-29, miR-342, miR-320, and miR-126 in exercise-induced cardiac protection.

Small EVs, or exosomes, are lipid bilayer vesicles with a diameter of 40–200 nm, secreted by most types of cells and existing in biological fluid such as bloods, urine, and saliva. Exosomes play important roles in cell-to-cell crosstalk, using their molecular cargo to communicate^61–63^. They are considered as a potential therapeutic strategy due to their immunologic inertia and stabilization^61^. MiRNAs in the exosomes play important roles in exercise-induced cardiac protection; known exosomes include miR-342-5p, miR-455, miR-320, miR-29, and miR-126. The mechanism of these exosomal-miRNAs in exercise-induced cardiac protection is shown in Fig. 4. Here in this section, we review function of exosomal-miR-342-5p in exercise-induced cardioprotection.

#### Exosomal-miR-342-5p

**Fig. 5:**
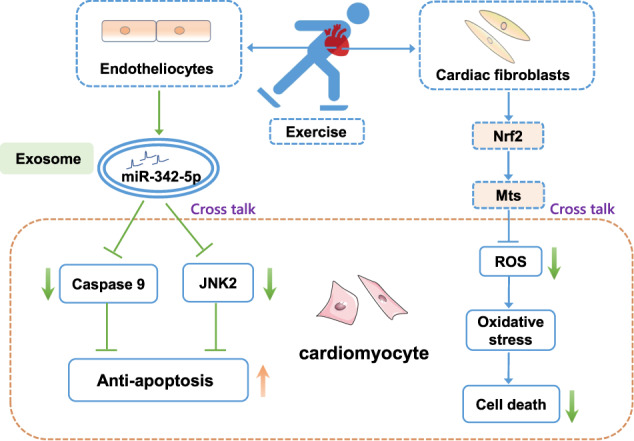
Crosstalk between noncardiomyocyte and cardiomyocytes in exercise-induced cardiac protection. Exercise promotes the expression of exosomal-nuclear factor-erythroid 2 related factor 2 (Nrf2)/metallothioneins (Mts) in cardiac fibroblasts, inhibiting cardiomyocyte reactive oxidative species (ROS) production and cell apoptosis. Endotheliocytes produce EVs (containing miR-342-5p) and deliver to cardiomyocyte, leading to an increase in the antiapoptosis capacity via inhibiting caspase 9 as well as c-Jun N-terminal kinase (JNK2).

MiR-342 is a mediator of exercise-induced cardiac protection. MiRNA sequencing reveals endothelial cell-derived exosomal-miR-342-5P undergoes a robust increase in exercised athletes and swam rats. Exercise-induced exosomal-miR-342-5P elevation improves the anti-apoptosis capacity of cardiomyocyte, mediated through inhibition of caspase 9 and cardiac c-Jun N-terminal kinase (JNK2)^64^ (Fig. 5). Also, a recent study in exercised healthy subjects showed that exercise dramatically induced 12 exosomal-miRNAs (miR-1-3p, -208a-3p, -486-5p, -23a-3p, -23b-3p, -451a, -16-5p, 378a-5p, -126-3p, -150-5p, -222-3p, and -186-5p) increased in the exosome fraction^65^.

### SIRT1 signaling

Exercise significantly increases SIRT1 expression^66^. SIRT1, one of the members of the sirtuin protein family, is an enzyme responsible for protein deacetylation, endoplasmic reticulum (ER) stress, oxidative stress, and energy metabolism. Increasing studies demonstrate that SIRT1 is involved in exercise-induced cardiac protection by alleviating adverse cardiac remodeling^67^ and reducing cell apoptosis^68^. Mechanistically, SIRT1 plays protective roles via (1) peroxisome proliferator-activated receptor-γ coactivator 1α (PGC-1α) activation, (2) eukaryotic initiation factor (eIF2α) pathway inactivation, and (3) forkhead boxo1 (FOXO1) reduction. First, SIRT1/PGC-1α signaling pathway activation can promote cell survival^69^. Exercise training upregulates SIRT1/PGC-1α/AMPK (AMP-activated protein kinase) and prolongs cell longevity^70^. Second, SIRT1 can attenuate ER stress-induced heart injury and cell apoptosis by interacting with eIF2α and inducing deacetylation of the lysine site (K141 and K143), which leads to protein kinase R-like endoplasmic reticulum kinase (PERK)/ eIF2α pathway inactivation^71^. Third, SIRT pathway activation regulates the deacetylation of FOXO1 and alleviates oxidative stress^72^.

### Nrf2-dependent pathway

Nrf2, a transcriptional regulator, has a central rols in antioxidative response by regulating more than 200 antioxidant genes such as NAD(P)H quinone oxidoreductase 1 (NQO1), glutathione-S-transferases (GSTs) and hemeoxygenase 1 (HO-1)^73^. When activated by stress and, notably, exercise, Nrf2 can translocate from cytoplasm to nucleus and trigger cytoprotective gene expression, thereby improving cardiac antioxidant capacity^74^ (Fig. 5). Mechanistically, ROS production promotes Nrf2 translocation and activation, leading to nuclear Nrf2 accumulation in the exercised mice heart. Consistently, one study reveals that exercise activates Nrf2 signaling in the exercised young person, thereby elevating the expression of antioxidant genes such as HO-1, NAD(P)H^75^. The content of Nrf2 activation is associated with the intensity of exercise, whose activation has different adaptions in different exercise protocols.

## The mechanism of exercise-induced cardiac protection

**Fig. 6:**
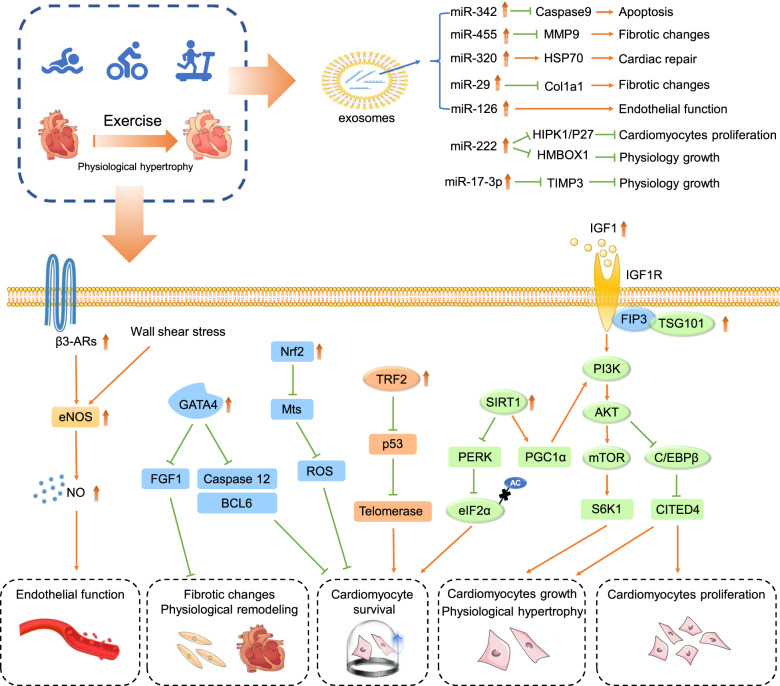
The targets identified from exercised hearts and their mechanism: killing multiple birds with one stone. In this section, we give an insight on exercise-induced cardioprotection, which includes (1) promoting cardiomyocytes proliferation; (2) facilitating cardiomyocytes growth and physiologic hypertrophy; (3) elevating the anti-apoptosis capacity of cardiomyocyte; (4) promoting vascular endothelial function; (5) inhibiting pathological remodeling and fibrosis; (6) promoting EVs production and exosomal-molecular transfer to cardiomyocytes.

Exercise induces several cellular adaptions, including proliferation and physiological hypertrophy of cardiomyocytes, antioxidation of myoblasts, production of endothelial NO, and a number changes in immunocytes. In this section, we review the mechanisms by which these adaptations are cardioprotective (Fig. 6).

### Exercise leads to cardiomyocyte growth and physiological hypertrophy

Myocardial hypertrophy is an adaptive response to growth factors and increased workload, and includes physiological and pathological hypertrophy. Studies exploring the effects of exercise on cardiac size and left ventricular function have demonstrated that exercise caused an increase in cardiac size and improvement in left ventricular diastolic function, which is physiological, rather than pathological, hypertrophy^76^. Physiological hypertrophy is a typical manifestation of the cardioprotective effects of exercise, quite different from pathologic hypertrophy^77,78^. The serum level of natriuretic peptides (N-terminal proatrial and probrain natriuretic peptide) are biomarkers, which allow differentiation between physiological and pathologic cardiac hypertrophy^79^. We propose that the molecular changes identified from exercise-induced physiological may be potential targets for prevention and treatment of CVDs. Mechanistically, the decreased fatty acid oxidation and increased glucose utilization are important molecular adaptations in response to increased workload^80^. We review important molecular alterations identified from exercise-induced physiological hypertrophic heart. Animal and clinical studies demonstrate that exercise training significantly improved physiological cardiomyocytes growth via activating the IGF1-PI3K/AKT pathway^81^, whose activation also improved the antiapoptotic capacity of cardiomyocytes. Exercise-induced IGF1-PI3K/AKT pathway activation is a crucial regulator of pressure overload-induced heart dysfunction^82^ and MI^83^. The activation of the IGF1-PI3K-AKT signaling pathway mediates exercise-induced cardiac physiological hypertrophy, whose activation may be a potential target for CVD therapy.

As one of the downstream targets of PI3K/AKT, C/EBPβ-CITED4 is also important in exercise-induced cardiac healthy growth. For example, a reduction in C/EBPβ mitigates the pressure overload-induced heart failure in mice^50,84^. In exercised mice, C/EBPβ deficiency inhibits NF-κB activation, thereby protecting cardiomyocytes from pathological hypertrophy^85^.

Functionally, physical exercise-induced physiological cardiac hypertrophy may compensate for the pathological myocardial remodeling such as MI^67,86–88^. A population study demonstrates that long-term endurance exercise caused cardiac remodeling not only in the left ventricle^87^, but also in the right^89,90^. Preclinical experiments demonstrates that voluntary exercise significantly promoted cardiac β_3_-Ars (β3-adrenergic receptor) expression, which is cardioprotective against ischemia reperfusion injury (IR/I). Physical exercise-induced cardiac β_3_-Ars elevation activates eNOS, which eventually stimulated NO production^91^. The β_3_-Ars-NO axis is a novel mechanism involves in exercise-induced cardiac healthy remodeling and protection.

### Exercise promotes cardiomyocytes proliferation

Mammalian cardiomyocytes have an extremely low proliferation rate^92^, which gradually decreases from 1 to 0.3% at the age above 70 years in human. The renewal of cardiomyocytes is no more than 50% over a lifetime^93,94^. Exercise promotes the capacity of cardiac proliferation in adult mice. In one study, two months of treadmill running increases the generation of cardiomyocytes^95^. Recently, Ana Vujic et al. used the multi-isotope imaging mass spectrometry (MIMS) to study cardiomyocyte proliferation and found ~4.6 folds of increase in adult exercised mice^95^.

Several molecules are involved in exercise-induced cardiomyocyte proliferation, such as C/EBPβ, miRs, and their downstream targets. Decreased C/EBPβ, or increased miR-222, both inhibit cardiac disorder via promoting proliferation and growth. C/EBPβ was the first transcriptional factor identified from adult exercised hearts, whose reduction has been associated with exercise-induced cardiomyocyte proliferation^50^. CITED4, which is downstream of C/EBPβ, contributed to exercise-induced cardiac protection. Exercise elevates GATA4, CITED4, and reduces C/EBPβ in MI mice, thereby protecting against cardiac disorder. Compared with the sham group, the MI mice exhibites a higher expression of C/EBPβ. Disruption of C/EBP expression in the adult heart inhibits MI-induced neutrophil infiltration and improved cardiac function^51,96^. CITED4 can attenuate cardiomyocyte apoptosis and autophagy flux after IR/I injury by activating the mTORC1 pathway^51,97^. Additionally, microRNAs, such as miR-222, miR-342, miR-17 are involved in exercise-induced cardiac proliferation. Cardiac miR-222 targets p27, HIPK1/2 and Hmbox1 and alleviates MI-induced injury.

Interestingly, exercise-induced cardiomyocyte proliferation may vary with age. To investigate this issue, different ages of rats (juvenile, adolescent and adult) are used in a recent study that underwent treadmill running for 4 weeks. Cell number analysis shows that the juvenile group has a significant increase in cardiomyocyte number (36%), higher than the adolescent and adult group. Exercise-induced cardiomyocyte proliferation is associated with the period of life^98^. Investigations in zebrafish also indicated that exercise training can promote cardiomyocyte proliferation^99^. To build the physiological hypertrophy model, adult zebrafish (~2.5 cm in body length) swam for sum up to 13 days, inducing an increase in cardiomyocytes proliferation^100^. As cardiomyocyte loss is a primary cause of CVD, such as in heart failure and myocardial infarction^92,101–103^, exercise-induced cardiomyocyte proliferation may be a potential preventative and therapeutic strategy for CVD.

Notably, the cell to cell crosstalk between cardiomyocytes and noncardiomyocytes plays important roles in cardiomyocyte proliferation^42^. Myofibroblasts, endotheliocyte and adipocytes can secret growth factors and cytokines and promote cardiomyocyte proliferation. In large animals, the isolated sinoatrial node cells (SANCs) from guinea pig heart can promote the generation of cardiomyocytes^104^, and may be an area of future study.

### Exercise improves the anti-apoptosis capacity of cardiomyocytes

Cardiomyocyte apoptosis is another cause of CVD^105,106^, which can be ameliorated by exercise intervention. In the treadmill exercise model, the apoptosis index (Bax/Bcl2, cleaved caspase 3/caspase 3) decreases after two months of running^107^. PI3K/AKT signaling inhibits cell apoptosis and promotes cardiomyocyte survival in exercise-induced cardiac function improvement in MI mice. Additionally, exercise inhibits TGFβ1 activation and leucocytes activation and migration, leading to an increase in anti-apoptotic capacity^86^. miR-222 inhibits TGFβ1 pathway (JNK, TGF-β receptor), which is cardioprotective against TAC or angiotensin II-induced remodeling and cardiac fibrosis^108^.

In the past decade, the association between telomerase activity and exercise training has received widespread attention. Voluntary running enhances the cardiac telomerase activity to ~2-fold of control, and caused an increase in TRF2 (telomere repeat binding factor 2) expression and p53 reduction^109^. Exercise also promotes the expression of telomere-protective genes and extends telomere length by activating the p38 MAPK pathway in myocardial cells^110^. Exercise attenuated stress-induced telomerase activity reduction and telomerase length shortening, which has been shown to prevent coronary heart disease^111^.

Furthermore, exercise diminishes cardiomyocyte apoptosis in an indirect way, including increased autophagy content and mitochondrial function. In doxorubicin-treated mice, endurance running exercise reduced dox-induced cell apoptosis by increasing the autophagy content containing increased LC3II and decreased p62, as well as mitochondrial autophagy^112^.

### Exercise improves vascular endothelial function

Endothelial cells play important roles in vascular homeostasis, whose dysfunction contributes to the pathological process of CVD. Aerobic exercise promotes NO production, balancing endothelium-dependent vascular homeostasis and regulating endothelial dysfunction in patients with heart failure^113^ and hypertension^114^. NO is produced by three kinds of nitric oxide synthase (NOS) enzymes: eNOS, neuronal nitric NOS (nNOS), and inducible NOS (iNOS)^115^. The activation of eNOS is associated with the endothelial function. Exercise training improves local blood flow by these means^116^ and coronary arteries vasodilation as well^117,118^. However, the mechanism of eNOS or NO upregulation after exercise is unknown. A recent study demonstrates that the change of wall shear stress (WSS) induced by exercise may contribute to the increase in NO production^119^.

MicroRNAs regulate endothelial function in exercised hearts and become a potential diagnostic biomarker for physical capacity^120^. Experimental evidence has revealed that exercise has a profound effect on the circulating miRNA profile. For example, exercise causes a significant increase in cardiac miR-492 expression in aortic endothelial cells and improves endothelial cell function^121^. Additionally, miR-126 induced by aerobic exercises inhibits the production of endothelium-derived factors such as NO and endothelin, eventually leading to further improvement in endothelial function^122^.

Of note, exercise-induced cardioprotective role of circulating microRNAs is not only through improving the endothelial function, but also in directly attenuating the progression of heart diseases. Specific exercise interventions such as Chinese Tai Chi can improve the life quality and block the progression of coronary heart disease (CHD) via inhibiting miR-24 and miR-155, whose level is lower in the serum of Tai Chi exercised CHD patients than that of sedentary ones^123^.

### Exercise inhibits fibroblast switch and cardiac fibrosis

Cardiac fibroblasts, or myofibroblasts, play integral roles in extracellular matrix degradation, repair, inflammation, and cardiac disease development^42^. Cardiac fibroblasts converting to myofibroblasts can lead to collagen deposition and tissue fibrosis, ultimately leading to pathological events^124^. Notably, cardiac fibroblasts are involved in exercise-induced cardiac growth due to their communication with cardiomyocytes^78^. To evaluate the effect of cardiac fibroblasts on exercise-induced cardiac growth, RNA sequencing is performed in exercised or diseased C57BL/6 mice. The transcriptional analysis demonstrates that antioxidant gene Nrf2 and metallothioneins (Mt, including Mt1 and Mt2) increase in exercised hearts, while they decrease in MI and TAC hearts. Furthermore, Mt1 and Mt2 deficiency exacerbates cardiac dysfunction in mice with MI. Mechanistically, the elevation of Mts in cardiac fibroblasts can transfer to cardiomyocytes, eventually inhibiting cardiomyocyte apoptosis^44^.

MiRNAs, such as miR-29, are a crucial mediator for exercise-induced physiological remodeling, leading to resistance to pathological myocardial remodeling^125^. MiR-29 significantly attenuates cardiac collagen deposition after MI injury via inhibition of cardiac collagen type I alpha 1 (ColIA1)^126^. Also, exercise attenuates cardiac fibrosis via activating the SIRT1/PGC-1α/PI3K/Akt pathway^67^.

Furthermore, running can protect the heart against cardiac fibrosis in female ovariectomized mice by reducing oxidative stress through 8-hydroxy-2’-deoxyguanosine, matrix metalloprotein 2 (MMP2) and collagen I/III reduction^127^. Of note, a low intensity of exercise still promotes the antioxidative activity of heart and improves the diastolic function of left ventricle, which is different from the function on right-ventricle^128^.

### Exercise promotes EVs production

Considered cargo in the body, EVs contain multiple molecule types including mRNA, microRNA, DNA, and proteins, mediating a wide variety of physiological and pathological processes^129,130^. Exercise induces the production of extracellular vesicles both in humans and experimental animals^131–133^. Of note, EVs are secreted into system circulation by almost all kinds of cells, including cardiomyocytes, endothelial cells, and platelets^40^. EVs deliver miRNA and proteins from endothelial cells or fibroblasts to cardiomyocyte cells in response to exercise, thereby having some role in regulation of cardiac function. In a diabetic heart model, the exercised heart tissues, as well as serum, are collected for isolation and analysis of EVs. A microRNA profile analysis showed that exercise significantly elevated miR-29b and miR-455 with respect to the sedentary group, inhibiting the expression of matrix metalloprotein 9 (MMP9) and cardiac remodeling^134^. Also, miR-320 in the EVs is potent in cardiac repair, whose presence improves hyperglycemia-induced cardiac injury via HSP20 pathway activation^135^. Exosomal-miR-126, produced by endothelial cells, increases after exercise^136^. EVs derived from these endothelial progenitor cells can promote vascular angiogenesis and protect heart function by delivering miR-126 in exercised hearts^137^. VEGF, the potential downstream target of miR-126, promotes the activation of AKT and ERK pathways, thereby improving cell survival^138^.

Additionally, EVs content differs greatly with and without exercise. Quantitative proteomic analysis is performed in EVs from the serum of exercised and rested human to figure out the protein cargo composition. Results showes that more than 300 proteins are transferred into system circulation^139^. The proteins enclosed in EVs are associated with several processes such as glycolysis (Glucose-6-phosphate 1-dehydrogenase elevation), which is activated to meet the energy requirements of exercise. As natural EVs and its contents play central roles in crosstalk between noncardiomyocyte and cardiomyocyte, EVs emerge as a potential therapeutic strategy for CVD.

### Exercise reduces metabolic disorders and transcriptional changes

**Fig. 7:**
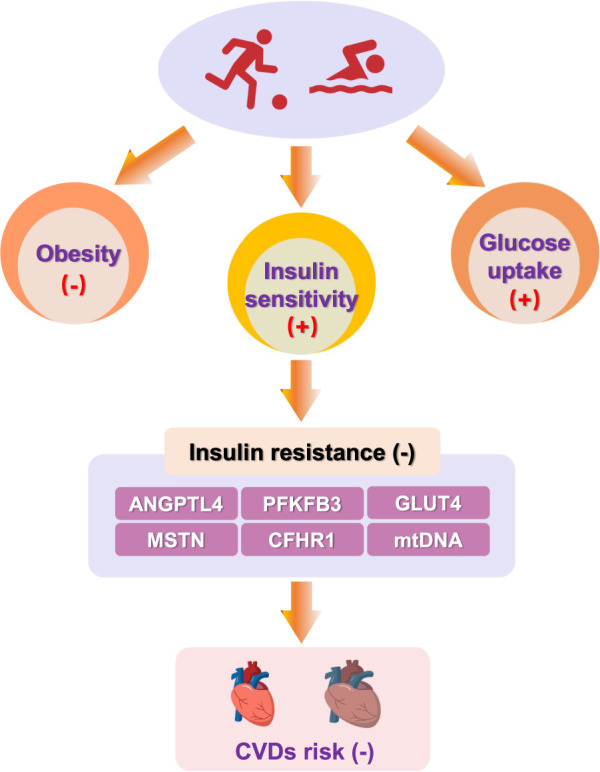
The effects of exercise on insulin resistance and transcriptional changes. Exercise improves insulin resistance via reducing obesity, improving insulin sensitivity, and glucose uptake.

With economic development, the burden of cardiometabolic risk rapidly expands and attracts much attention. Cardiometabolic disease-induced mortality including insulin resistance, type 2 diabetes mellitus, and obesity, are increasing. Wang et al., reportes a significant increase in cardiometabolic risk among Chinese adults in a recent study^140^. Obesity and elevated dyslipidemia in younger populations implies an increasing potential of cardiometabolic diseases. Aerobic exercise, as we have seen, can ameliorate these effects, improving insulin resistance^28^, glucose level^141^ and overweight^142^ and reduce the cardiometabolic risk^143,144^. Exercise-induced transcriptional changes are important to reducing insulin resistance, partially accomplished through the transcription of insulin resistance-related genes and mitochondrial DNA (mtDNA) (Fig. 7).

Studies exploring transcriptional changes induced by exercise in skeletal muscle have shown that genes related to muscle growth and antiatrophy are significantly remodeled. Among these, ANGPTL4 (angiopoietin like 4) is regulated by exercise, whose gene expression is associated with DNA methylation^145^. Hu et al. analyzes gene profiles and transcriptional changes induced by exercise in resistant patients and healthy controls with exercise training. Differentially expressed genes are analyzed and reveals that alteration of insulin resistance-related genes, such as MSTN (myostatin), CFHR1 (the complement factor H related 1), PFKFB3 (6-phosphofructi-2-kinase/fructose-2,6-biphosphatase) were reversed by exercise^146^. In addition, transcriptional changes of genes associated with glucose metabolism are investigated during exercise, ultimately demonstrating improved glucose uptake and oxidation by increasing glucose transporter 4 (Glut4) transcription in a AMPKα-independent manner^147^.

Mitochondrial DNA (mtDNA) is also associated with insulin sensitivity. Tomas et al. report that insulin resistance is associated with decreased mtDNA content in adolescents^148^. Additionally, mtDNA copy number is considered a biomarker of glucose homeostasis and insulin sensitivity in nondiabetic woman^149^. Cytochrome b (Cytb) is one of these genes encoded by mtDNA, and is decreased in type 2 diabetic mice when compared to control. The dyslipidemia-induced Cytb mRNA reduction, in one study, is rescued by aerobic exercise^150^.

## Conclusions and perspectives

In this review, we examine the cardiac benefits of exercise and summarize the key factors, and their mechanisms, in exercise-induced cardiac protection, including IGF1-PI3K/AKT signaling, C/EBP-β-CITED4, cardiac miRNAs (miR-222, miR-17-3p, etc.), exosomal-miRNAs (miR-342, miR-29, etc.), SIRT1 and Nrf2-Mts. Furthermore, we review the role of noncardiomyocytes in exercise-induced cardioprotection, including cardiac fibroblasts and endothelial cells. These cardioprotective mechanisms include the following: (1) PI3K/AKT activation which reduces C/EBP-β expression and causes CITED4 and GATA upregulation after exercise training. This ultimately leads to an increase in antiapoptotic activity and enhancement of physiological hypertrophy. (2) Increased miR-222 induces physiological hypertrophy of cardiomyocytes via inhibiting HMBOX1 expression. MiR-222 mimic supplement can promote cardiomyocyte proliferation via suppression of HIPK1/p27. (3) miR-17-3p targets TIMP3, activates EGFR/JNK/SP-1, and regulates PTEN/AKT pathway, leading to cardiomyocyte cell survival. (4) Exosomal-miR-342, produced by endotheliocytes and delivered to cardiomyocytes, can attenuate cardiomyocyte apoptosis via inhibition of caspase 9 and JNK2. (5) SIRT1 signaling promotes the antiapoptotic capacity of cardiomyocytes via inhibition of p53, oxidative stress, and eIF2α acetylation. (6) Nrf2/Mts expression, in fibroblasts, induces Mts translocation to cardiomyocytes, thereby suppressing oxidative stress in cardiomyocytes. In conclusion, targets identified from exercised heart may have therapeutic potential in cardioprotection in CVDs, ‘killing multiple birds with one stone’ (Fig. 6).

## Data Availability

The data that support the findings of this study are available from the corresponding author upon reasonable request.
